# Complexes of Ectoine with the Anionic Surfactants as Active Ingredients of Cleansing Cosmetics with Reduced Irritating Potential

**DOI:** 10.3390/molecules25061433

**Published:** 2020-03-21

**Authors:** Tomasz Bujak, Martyna Zagórska-Dziok, Zofia Nizioł-Łukaszewska

**Affiliations:** Department of Technology of Cosmetic and Pharmaceutical Products, University of Information Technology and Management in Rzeszow, Kielnarowa 386a, 36-020 Tyczyn, Poland; mzagorska@wsiz.rzeszow.pl (M.Z.-D.); zniziol@wsiz.rzeszow.pl (Z.N.-Ł.)

**Keywords:** ectoine, irritant potential, cosmetics, safety of use

## Abstract

For many years, an increasing number of diagnosed atopy and skin problems have been observed. For people affected by the problem of atopy, the selection of skin care products, including cosmetics, is extremely important. Cleansing cosmetics, due to their ability to cause skin irritations and disturb the hydrolipidic barrier, can increase problems with atopic skin. New solutions to reduce the effects of these products on the skin are very important. In this work, the effect of ectoine on the properties of anionic surfactants was analyzed. Based on model systems, analysis of the effect of ectoine on the irritating effect of four anionic surfactants and their ability to solubilize model sebum was performed. Antioxidant activity was also evaluated, and cytotoxic studies were performed on cell cultures. It was shown that the addition of ectoine to the anionic surfactant solutions improves its safety of use. After introducing ectoine to the surfactant solution, a decrease of irritant potential (about 20%) and a decrease in the ability to solubilize of model sebum (about 10–20%) was noted. Addition of ectoine to surfactant solutions also reduced their cytotoxicity by up to 60%. The obtained results indicate that ectoine may be a modern ingredient that improves the safety of cleansing cosmetics.

## 1. Introduction

The skin is a barrier separating the body from the external environment. It serves very important functions for our body. In addition to protecting the body against water loss and infections of microorganisms, it plays an important cosmetic role. The development of medical sciences and work on improving skin care methods have resulted, among other things, in the use of ectoine in products for daily skin care, both in healthy people and people with atopic skin or other diseases [1,2,3].

Ectoine is a rare amino acid, 1,4,5,6-tetrahydro-2-methyl-4-pyrimidine carboxylic acid of an amphoteric nature, produced by bacteria living in harsh environments called extremophiles. This substance is one of the osmoprotectants, synthesized by microorganisms in order to protect against various types of environmental stress. Studies have shown that ectoine protects epithelial cells, stabilizes cell membranes, moisturizes the skin and reduces inflammation [3,4,5,6].

Ectoine and its derivatives limit the action of hydroxyl radicals and also reduce the oxidative damage to mitochondrial DNA in human skin fibroblast cells caused by UVA radiation. Moreover, they exhibit antioxidant activity on protein structures. Studies have shown that in the free radical oxidation process ectoine can act protecting on the lactate dehydrogenase. Due to the strong water binding activity in the cell and the protective effect on protein cell structures, ectoine can be particularly useful in preventing the drying of the skin, which can lead to its premature aging. This compound prevents water loss from cells, regulates their turgor without interfering with metabolism. The moisturizing effect of ectoine, as well as its protective effect on skin cells in case of sudden stress caused by lack of water, have also been demonstrated. Research conducted by Graf et al. has shown that ectoine reduces transepidermal water loss (TEWL) and has a short-lasting moisturizing effect on the skin [1,2,3].

Ectoine is used more and more often as an active ingredient in cleaning agents. Frequent use of cleaning agents destroys the lipid layer and accelerates skin aging. It has been shown that ectoine and its derivatives play a protective role during the washing process. They contribute to the stabilization of intracellular proteins, improve the hydration of hydrophobic macromolecules and have a protective effect on the cell membrane [1,7].

Due to its high effectiveness, ectoine can be broadly applied in cosmetics where it can be added as an active ingredient to cleansing products, moisturizing creams or products that protect the skin against UV radiation and the effect of premature skin photoaging [2,8,9,10].

In this study, an attempt was made to assess the biochemical and cytotoxic properties of ectoine and determined its effect on the irritating potential of prototype body wash gels. The antioxidant potential of the tested products was determined and their effect on the proliferation of keratinocytes and fibroblasts was assessed. In addition, the formula for a natural body wash gel was developed. For each of the preparations, the irritant potential was determined by determining the zein test and testing the solubility of model sebum.

## 2. Result and Discussion

### 2.1. Determination of Irritant Potential

The most disadvantage of using cleansing cosmetics is their ability to cause skin irritations. Surfactants—the main ingredients of this product group—are responsible for this process [11,12,13,14,15,16,17,18,19,20,21,22,23,24,25]. Anionic surfactants have the strongest ability to cause irritations because they bind to protein molecules through strong ionic bonds. On the other hand, nonionic surfactants have the lowest ability to cause skin irritations. They interact with skin protein molecules through weak hydrogen bonds [11,12,13,14,15,16,19,20,21,22,23,24,25,26]. The interaction of surfactants with skin proteins is only one of the mechanisms of skin irritation postulated in the literature, but these interactions play a major role in this process. Surfactants can also interact with a components of intercellular lipids and sebum, as well as with live skin cells. The irritant potential of surfactants should be considered in all of these areas [11,12,13,14,15,16].

**Figure 1:**
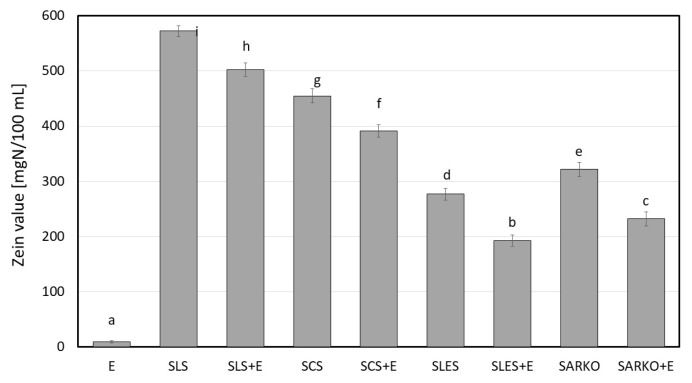
Irritant potential (zein value) of 2.5 wt.% ectoine (E), 1 wt.% anionic surfactant solutions sodium lauryl sulfate (SLS), sodium coco sulfate (SCS), sodium laureth sulfae (SLES), sodium lauroyl sarcosinate (SARKO) and 1 wt.% surfactants solutions combined with 2.5 wt.% of ectoine. Different letters on the charts indicate significant differences between groups (*p* < 0.05), number of repetitions: 5.

**Figure 2:**
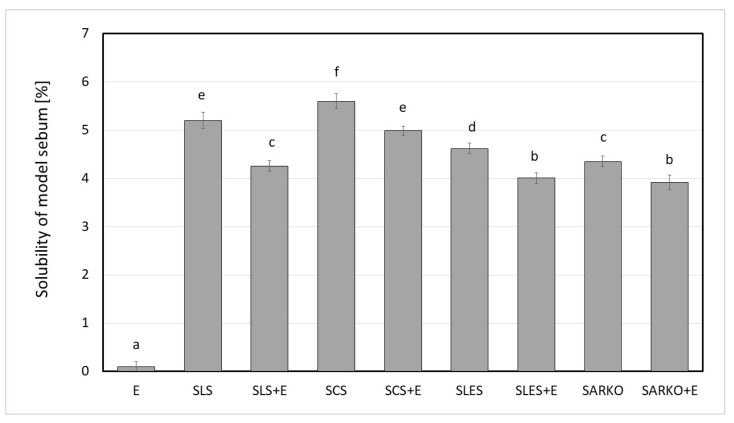
Ability to solubilization of model sebum of 2.5 wt.% ectoine (E), 1 wt.% anionic surfactant solutions (SLS, SCS, SLES and SARKO) and 1 wt.% surfactants solutions combined with 2,5 wt.% of ectoine. Different letters on the charts indicate significant differences between groups (*p* < 0.05), number of repetitions: 5.

**Figure 3:**
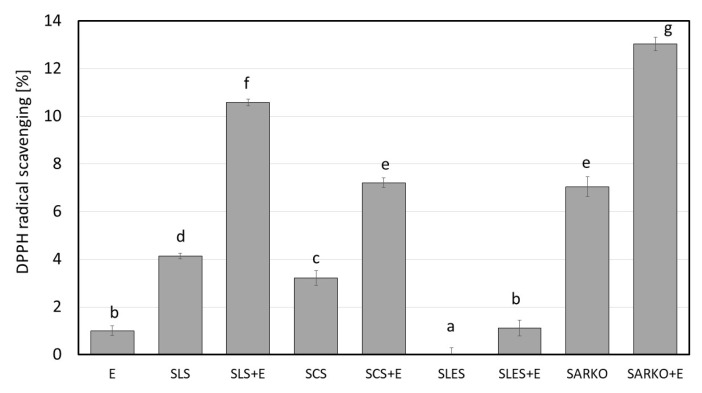
Antioxidant potential of 2.5 wt.% ectoine (E), 1 wt.% anionic surfactant solutions (SLS, SCS, SLES and SARKO) and 1 wt.% surfactants solutions combined with 2.5 wt.% of ectoine. Different letters on the charts indicate significant differences between groups (*p* < 0.05), number of repetitions: 5.

**Figure 4:**
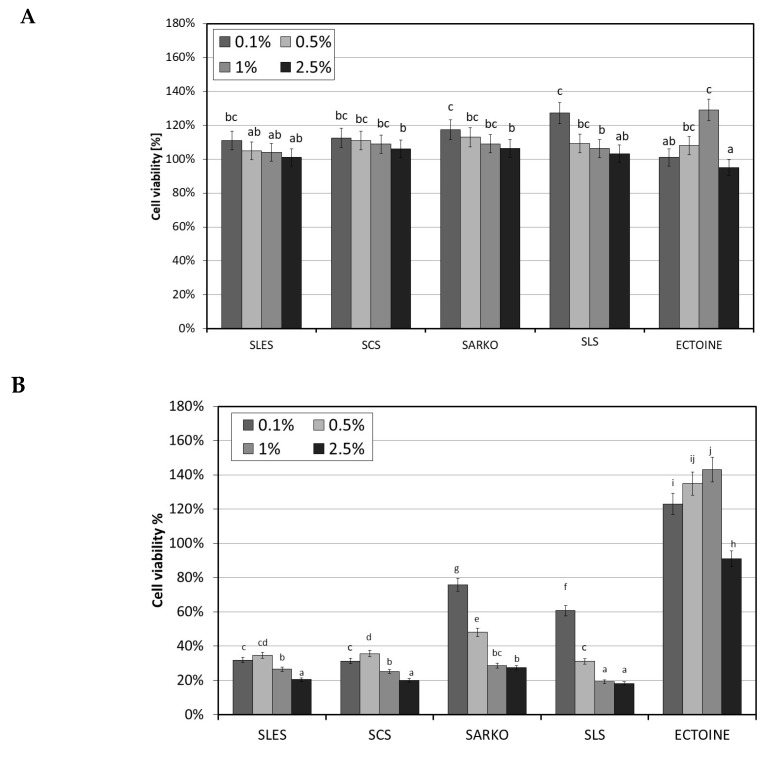
The effect of different concentrations (0.1%, 0.5%, 1% and 2.5%) of four types of surfactants (SLES, SCS, SARKO and SLS) and ectoine on neutral red dye uptake in cultured fibroblasts after 1 (**A**) and 24 (**B**) hours of exposure. Data are the mean of four independent experiments, each consisting of three replicates per treatment group. Different letters on the charts indicate significant differences between groups (*p* < 0.05), number of repetitions: 12.

**Figure 5:**
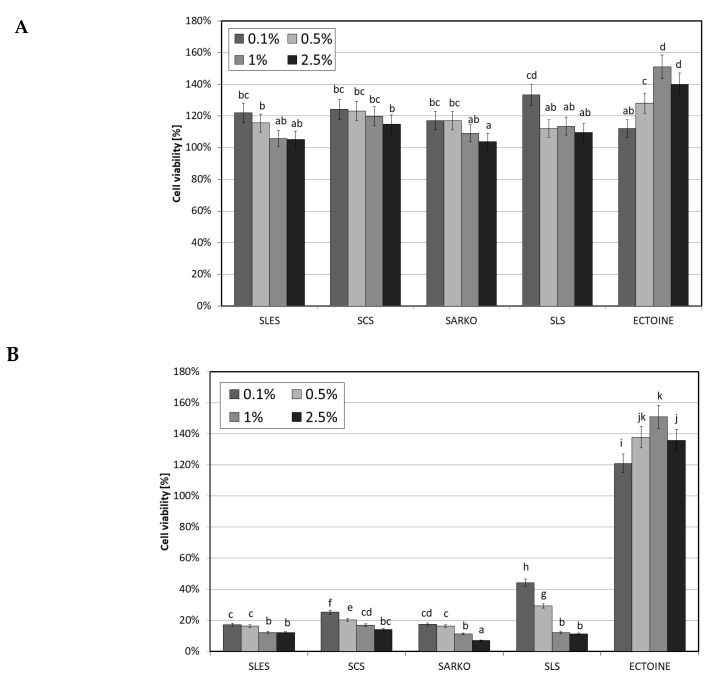
The effect of different concentrations (0.1%, 0.5%, 1% and 2.5%) of four types of surfactants (SLES, SCS, SARKO and SLS) and ectoine on neutral red dye uptake in cultured HaCaT cells after 1 (**A**) and 24 (**B**) hours of exposure. Data are the mean of four independent experiments, each consisting of three replicates per treatment group. Different letters on the charts indicate significant differences between groups (*p* < 0.05), number of repetitions: 12.

**Figure 6:**
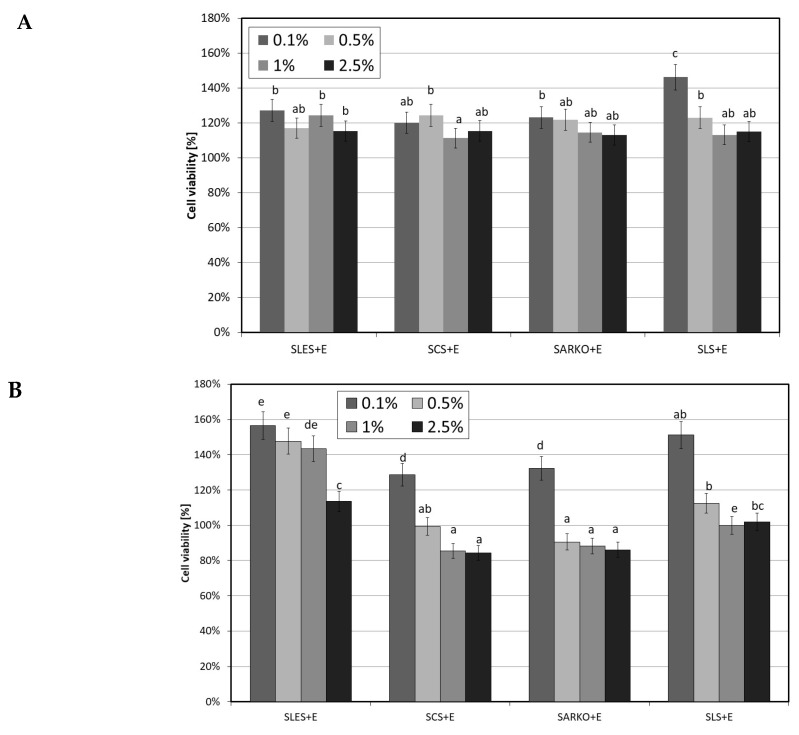
The effect of different concentrations (0.1%, 0.5%, 1% and 2.5%) of four types of surfactants (SLES, SCS, SARKO and SLS) combined with 2.5% ectoine (E) on neutral red dye uptake in cultured fibroblasts after 1 (**A**) and 24 (**B**) h of exposure. Data are the mean of four independent experiments, each consisting of three replicates per treatment group. Different letters on the charts indicate significant differences between groups (*p* < 0.05), number of repetitions: 12.

**Figure 7:**
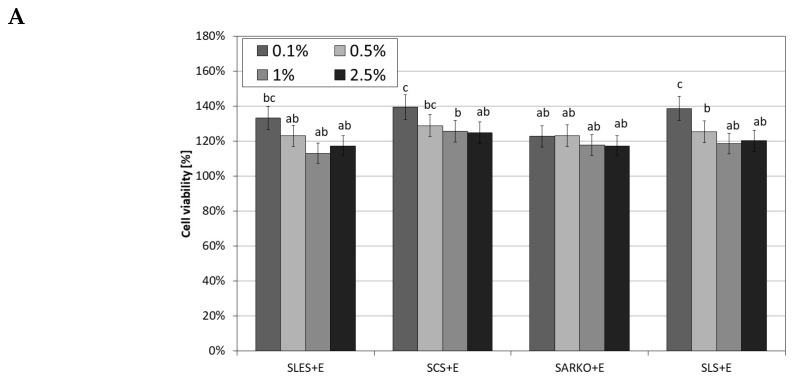

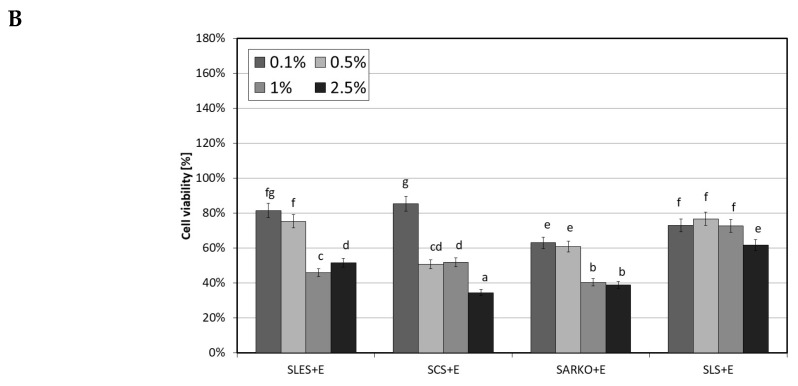
The effect of different concentrations (0.1%, 0.5%, 1% and 2.5%) of four types of surfactants (SLES, SCS, SARKO and SLS) combined with 2.5% ectoine (E) on neutral red dye uptake in cultured HaCaT cells after 1 (**A**) and 24 (**B**) hours of exposure. Data are the mean of four independent experiments, each consisting of three replicates per treatment group. Different letters on the charts indicate significant differences between groups (*p* < 0.05), number of repetitions: 12.

In this study, the effect of the addition of ectoine to 1 wt.% aqueous anionic surfactant solutions on their irritant potential was analyzed. Zein number (or zein value) analyses (interaction of surfactants with skin proteins), the ability to solubilize of model sebum (interaction with lipids) and cytotoxicity studies (interaction with skin cells) were performed for all analyzed systems. In addition, antioxidant properties were determined. The results are presented on Figure 1, Figure 2, Figure 3, Figure 4, Figure 5, Figure 6 and Figure 7.

A significant decrease in the zein value (ZV) was observed after adding ectoine to model anionic surfactant solutions (*p* < 0.05). The highest values were obtained for sodium lauryl sulfate (SLS) and sodium coco sulfate (SCS) solutions (572 and 455 mg N/100 mL, respectively), while lower values of ZV were observed for sodium laureth sulfate (SLES) and Sodium lauroyl sarcosinate (SARKO) solutions (276 and 321 mg N/100 mL). These results are consistent with the literature data [11,12,13,14,15,16,17,18,19,20,21,22,23,24,25,26,27,28,29]. Surfactant solutions with ectoine are characterized by much lower values of the zein value (ZV). In the case of sodium lauryl sulfate + ectoine and sodium coco sulfate + ectoine solutions (SLS + E and SCS + E, respectively), the decrease of the irritant potential was about 12% and 20%, respectively. A much higher ability of ectoine to reduce ZV was observed for sodium laureth sulfate + ectoine and sodium lauroyl sarcosinate + ectoine (SLES + E and SARKO + E) solutions. For these systems, the decrease of the irritant potential was about 30% compared to solutions without ectoine. According to the literature data [12,14,20,21,22,23,24,25,26] the irritant potential of cleansing cosmetics can be predicted from zein value. If the value is above 400 mg N/100 mL-, cosmetics are classified as strongly irritant to the skin; in the range of 200–400 mg N/100 mL-, moderately irritant; and below 200 mg N/100 mL, non-irritant.

Surfactants–skin protein interactions are indicated as the main mechanism leading to skin irritations. The strongest ability to bind with proteins is demonstrated by single molecules of surfactants (monomers). It is confirmed by the results, which indicate that the strongest adsorption of surfactants with protein molecules occurs before reaching the critical micellization concentration (CMC) [12,14,15,16,27,28,29]. The power of irritant potential is proportional to the type of surfactant, molecule structure, surfactant concentration and the contact time with surfactants [11,12,13,14]. After exceeding CMC, surfactant micelles formed in the solution have a significantly lower irritant potential. However, surfactant monomers released from the micelle structures may induce the occurrence of irritation. Only micelles with very small sizes (as in the case of SLS) can penetrate the skin pores and show high irritating potential [11,12,13,14,15,16]. As the literature data show, the reduction of the irritant potential of anionic surfactants occurs after the introduction of polymers, protein hydrolysates, plant extracts and other types of surfactants, e.g., amphoteric, cationic or nonionic into their solutions [11,12,13,14,15,16,17,18,19,20,21,22,23,24,25,26].

As an amphoteric amino acid, ectoine will be able to interact with micelles formed in solutions [1,2,3,30]. Much literature data show that surfactant–amino acid interactions reduce the CMC of surfactants and increase the size of micelles formed in the solutions [27,28,29]. Changes in these parameters cause an increase in the stability of the micelles. The number of monomers released from the micelles decreases, and thus the irritant potential of surfactants is reduced (lower number of the monomers is noted in the bulk phase of the solution) [11,12,13,14,27,28,29]. Under conditions of our study (acid solutions), ectoine will be in the cationic form. Incorporation of this form of ectoine (strongly hydrated by water molecules) into the micelle structure will additionally stabilize the micelles of anionic surfactants by increasing the interaction of hydrophilic parts of these compounds and increasing the size of micelles. The protein–ectoine and surfactant–ectoine interactions have been previous studied [1,2,3,4,31]. The presence of ectoine in protein solutions causes the formation of a hydration shell on their surface. In addition, the volume occupied by protein molecules is reduced, which reduce the contact surface between proteins and solution components [1,2,3,7]. The reduction of irritant potential in the surfactant–ectoine solutions will be also caused by the protective effect of ectoine on zein. Reducing the contact surface of the protein molecule with the surfactant molecules will reduce the solubilization of the protein in ectoine-surfactant solutions.

In the next stage of research, the ability to solubilize model sebum by model systems was assessed. It was shown that the addition of ectoine to solutions of anionic surfactants slightly reduces their ability to remove sebum from the skin surface and solubilize intercellular lipids components. Statistically significant differences (*p* < 0.05) were noted for SLS, SCS as well as SARKO and SLES, for which the solubilization ability differed slightly. The value of the analyzed parameter was at the level of 4.5–5.5% and the power of the analyzed anionic surfactants to solubilization of the model sebum can be represented by: SCS > SLS > SLES > SARKO. The introduction of ectoine into surfactant solutions reduces the solubilization ability from about 10% (SARKO + E, SLES + E) to about 20% (SLS + E), in relation to surfactant alone solutions. The ability to solubilization of sebum and dissolve the components of the intercellular lipid components is one of the mechanisms potentially leading to skin irritation. Removal of sebum from the skin surface and change in the structure of intercellular lipid increase the amount of water removed from the surface layers of the epidermis (TEWL). In turn, the increase in TEWL leads to a disorder of the skin’s barrier function, allowing penetration into deeper layers of the epidermis of a various types of factors that may cause irritation [11,12,13]. Ananthapadmanabhan et al. [32,33] have been shown that the composition of surfactants in the formulations of a cleansing cosmetics significantly affects the ability of the system to damage the hydrolipidic barrier. For example, SLES solutions containing of amphoteric surfactant cocamidopropyl betaine (2:1 ratio) have a stronger ability to dissolution of the skin lipids than the solution of SLS [32,33]. On the other hand, the addition of cationic amphiphilic polymers reduces the solubilization ability of anionic surfactants [26].

Free radicals are important factors that may influence the change in the structure of intercellular lipids, and thereby damage the barrier function of the skin. They may lead to irritations or skin inflammations [11,12,34,35]. Free radicals have the ability to oxidize components of intercellular lipid and sebum, especially cholesterol, squalene and other unsaturated compounds. Products of the lipid oxidation process cause damage of the liquid crystal structure of the skin leading to a decrease in its integrity and liquefaction. Reduction of the skin barrier function results in an increase of TEWL and leads to easier penetration of an irritant factors into deeper layers of the skin. The protective effect of ectoine in combination with SLS has been previously studied [1,2,3,4,10,30]. The addition of ectoine to the SLS solution was shown to have a strong protective effects, minimizing cell damage and reducing the TEWL or drying potential of the surfactant [1,2,3,4,10,17,24,34,35].

In this study, the antioxidant ability of the analyzed anionic surfactants, ectoine aqueous solutions and ectoine solutions with surfactants was determined. The obtained results indicate that both ectoine as well as SLS and SCS solutions do not show antioxidant potential towards the DPPH radical (around 1%). For the SLES solution, a pro-oxidative effect was found. Only for the SARKO solution, an antioxidant potential of around 8% was noted. Sarcosinates are surfactants obtained from sarcosine—an amino acid—which has antioxidant activity [12,14]. The addition of ectoine to the surfactant solutions significantly increased the antioxidant activity of surfactant solutions by about 200–300% in relation to the surfactant alone solutions. A mechanism characteristic for polyphenolic compounds should be expected (formation of a more stable radical of the antioxidant molecule and destabilization of the reactive DPPH radical). Activation of the ectoine molecules by increase of its hydrophobically in the ectoine-surfactant complexes solution may lead to increase of antioxidant activity of ectoine. Literature data show that increase of hydrophobically of amino acids leads to increase of antioxidant properties [36,37,38,39,40,41,42,43,44].

### 2.2. Cytotoxicity

Analysis of the cytotoxic effect of the tested surfactants on human skin cells—fibroblasts and keratinocytes demonstrated differences in the toxic effects between individual surfactants. The obtained results clearly showed that the time of cell exposure to surfactants has a key impact on the effect of their action. The conducted assays indicated that the tested surfactants in the applied concentration range (0.1–2.5%) after 1 h incubation stimulated both keratinocyte and fibroblast activity. The increase in cell metabolic activity was greatest for SLS and was about 30% for both cell types at a concentration of 0.1%. Higher surfactant concentrations also stimulated cell activity, but to a lesser extent. As the surfactant concentration increased, cell activity decreased (Figure 4A and Figure 5A). Different results were observed after 24 h of exposure of test cells to surfactants. After this time, a significant cytotoxic effect was observed on both cell lines for all four types of surfactants. The results of the conducted analyses indicate that the tested compounds show slightly higher toxicity towards HaCaT cells than fibroblasts. In the case of fibroblasts, the weakest cytotoxic activity was demonstrated by SARKO and SLS, while in the case of HaCaT cells the weakest effect was shown by SLS. Similarly to 1 h incubation, the cytotoxic effect closely depended on the concentration of surfactant and increases with increasing concentration. A particularly strong cytotoxic effect was observed in the case of keratinocytes, in which all surfactants used except SLS resulted in an over 80% decrease in the metabolic activity of cells (Figure 4B and Figure 5B).

As part of the study, the effect of the compound of natural origin—ectoine—was also examined. The results of experiments assessing the effect of its various concentrations (0.1–2.5%) on both cell lines show that this compound has a positive effect on the viability of both fibroblasts and keratinocytes. All the tested concentrations increased the metabolic activity of these cells. As part of the study, an assessment was also made of the appropriateness of using a combination of surfactants and ectoine. Thus, the viability of both cell lines treated with test surfactants in combination with 2.5% ectoine was tested. The ectoine concentration was selected on the basis of research showing the positive impact of this concentration and the recommendations of cosmetics manufacturers regarding the concentration of this substance in cosmetics intended for people with atopy problems. Results obtained indicate that ectoine significantly reduces the cytotoxic effect of surfactants on skin cells. The increase in cell viability compared to the use of surfactants alone after 1 h incubation was about 10–15% (Figure 6A and Figure 7A). A strong effect of reducing the cytotoxic activity of the tested surfactants by using 2.5% ectoine was observed after 24 h of incubation of cells with surfactants. In the case of analyses carried out on fibroblasts, ectoine was shown to be able to increase the viability of these cells by up to 120% at SLS concentrations below 1%. The use of this compound eliminated the cytotoxic effect of all SLES and SLS concentrations tested and the lowest SCS and SARKO concentrations tested (Figure 6B).

In the case of keratinocytes, which turned out to be more sensitive to the effect of surfactants, the addition of 2.5% ectoine also significantly reduced their cytotoxic effect. A particularly strong effect was observed for SLES and SCS, where the increase in cell activity reached up to 60% (Figure 7B).

Skin cells, both keratinocytes and fibroblasts, are heavily exposed to surfactants present in cosmetic preparations. According to literature reports, their long-term use causes skin irritation and leads to various damages to its structure. These agents may affect the enzymatic activity, which may result in various disorders of the function and structure of this organ [45]. In our previous studies, using SLES and SCS, we have shown that anionic surfactants at low concentrations and after a short incubation time can stimulate the metabolic activity of HaCaT cells, while higher concentrations and longer exposure times have a cytotoxic effect [20]. The same results have been confirmed for all four compounds tested in this study, both in the case of keratinocytes and fibroblasts. The stimulating effect of low SLS concentrations (below 0.06 mg/mL) on the growth of fibroblasts was also confirmed by Benoit et al. who pointed to the concentration-dependent cytotoxic effect of this agent [46]. High SLS toxicity to keratinocytes has been demonstrated by Bigliardi et al. who showed toxic effects on keratinocytes above 3 µg/mg and a proproliferative effect at low concentrations of this anionic surfactant [47]. Literature data indicate that low concentrations of free radicals can increase cell proliferation and metabolic activity by activating cellular defense and repair mechanisms [48,49]. Due to the fact that Mizutani et al. demonstrated that anionic surfactants such as SLS can stimulate the production of reactive oxygen species, the increase in the proliferation of keratinocytes and fibroblasts treated with the surfactants may result from the production of small amounts of free radicals by these cells as a result of their exposure to these agents [50]. The decrease in the activity of the tested cells caused by the longer exposure time and higher concentrations of the analyzed surfactants may be due to the formation of a large number of free radicals, which causes a cytotoxic effect [51]. The effect of the tested surfactants on cells is most often associated with the formation of ionic bonds between cell membranes and anionic surfactants. The result is the formation of structural changes in the plasma membrane and conformational changes of membrane proteins. Perhaps lipids, which are an extremely important component of cell membranes, are also involved in this process [52]. Other authors report that inactivation of cell oxidative functions by surfactants such as SLS may be due to changes in cell surface charge [53]. Damage to cells exposed to anionic surfactants, e.g., SLS, is associated with their cytolytic effect [54]. It should be noted here that the effect of surfactants largely depends on their chemical structure, the length of the surfactant tail as well as the nature of the charged head group [52]. Probable lytic effect on cells is associated with the adsorption of surfactant on the surface of the membrane and its penetration. This results in changes in molecular organization that contribute to changes in membrane permeability [52,55]. The reduction of the cytotoxic effect of the tested surfactants by the use of ectoine may result from the fact that it can accumulate within the cells without interfering with the cellular processes. It is also able to protect cells against oxidative and osmotic stress, which can stimulate proliferation and increase metabolic activity of cells, e.g., by protecting cell membranes and stabilizing enzymatic activity [1,2].

## 3. Materials and Methods

### 3.1. Materials

Raw materials used in the commercial cosmetic products were used to develop the body wash gels: sodium laureth-2 sulfate (trade name Brensurf 25; supplier Brenntag, Warsaw, Poland), sodium lauryl sulfate (Rosulfan L, PCC Exol, Brzeg Dolny, Poland), sodium coco sulfate (Sulfopon C1216, BASF, Ludwigshafen, Germany), sodium lauroyl sarcosinate (Crodasinic LS30, Croda, Snaith, Great Britain), Ectoin (RonaCare Ectoin, Merck, Darmstadt, Germany), citric acid (citric acid, Chempur, Piekary Śląskie, Poland) and Milli-Q water. The formulations of analyzed solutions is presented in Table 1. A representative concentration of 1 wt.% of surfactants was selected for the study. Cleansing cosmetics available on the market contain about 10–15% surfactants, but they are diluted with water during the cleaning process.

In the physico-chemical tests were used: zein from corn (zein, Sigma Aldrich, Saint Louis, MO, USA), potassium sulfate (Chempur, Piekary Śląskie, Poland), copper sulfate pentahydrate (Chempur, Piekary Śląskie, Poland), sulfuric acid 98% (Chempur, Piekary Śląskie, Poland), Tashiro indicator (Chempur, Piekary Śląskie, Poland), sodium hydroxide, citric acid (Chempur, Piekary Śląskie, Poland). All reagents were analytical grade.

### 3.2. Methods

#### 3.2.1. Solubility of Model Sebum

Five grams of stearic acid and 0.5 g of a mixture of cholesterol and ceramides (Bio-Ceramidyl Pure) was mixed with 100 mL of samples. The mixtures were shaken on a shaker with water bath (24 h at 25 °C). After 24 h the solutions were filtered on Whatman No. 1 filters. The filters were then washed repeatedly with demineralized water and dried. The amount of solubilized stearic acid was determined gravimetrically [32,33]. The values presented in the figures represent average values obtained from five independent measurements.

#### 3.2.2. Determination of Irritant Potential–Zein Volume

Irritant potential of the products was measured using zein test [27,28,29]. In a solution of surfactants, the zein protein denatures and then dissolves in the solution. This process simulates the behavior of surfactants towards skin proteins.

To 40 mL of samples (surfactants and mixtures of surfactants with ectoine), 2 ± 0.05 g of zein from corn was added. The solutions with zein were shaken in a shaker with water bath (60 min. at 35 °C). The solutions were filtered on Whatman No. 1 filters and then centrifuged at 5000 rpm for 10 min. The nitrogen content in the solutions was determined by Kjeldahl method. One milliliter of the filtrate was mineralized in sulfuric acid (98%) containing copper sulphate pentahydrate and potassium sulphate. After mineralization, the solution was transferred (with 50 mL of Milli-Q water) into the flask of the Wagner–Parnas apparatus. In the next step, 20 mL of sodium hydroxide (25 wt.%) was added. The released ammonia was distilled with steam. Ammonia was bound by sulfuric acid (5 mL of 0.1 N H_2_SO_4_) in the receiver of the Wagner–Parnas apparatus. The unbound sulfuric acid was titrated with 0.1 N sodium hydroxide. Tashiro solution was used as an indicator. The zein number (ZN) was calculated from the equation:

ZN = (10 − V1)∙100∙0.7 [mg N/100 mL](1)

where V1 is the volume (cm^3^) of sodium hydroxide used for titration of the sample. The final result was the arithmetic mean of five independent measurements.

#### 3.2.3. DPPH Radical Scavenging Assay

The antioxidant activity of the model solutions (surfactants and mixtures of surfactants with ectoine) was analyzed using DPPH free radical scavenging assay, according to the method described by Brand-Williams et al. [11]. 100 µL of 4 mM ethanol solution of DPPH was mixed with 100 µL of analysis samples (1% aqueous solutions of surfactants and 1% aqueous solutions of surfactants with 2.5% of ectoine). The absorbance was measured at λ = 516 nm after 30 min using UV-Vis spectrophotometer Filter Max 5 (Thermo Scientific). DPPH solution mixed with equal volume of distilled water was served as a control. The percentage of the DPPH radical scavenging were calculated using the equation:

%DPPH• scavenging = [Abs control−Abs sample]/Abs control × 100%(2)

The final result was the arithmetic mean of five independent measurements.

#### 3.2.4. Cell Culture

Cytotoxicity studies of the tested surfactants and ectoine were performed on two cell lines: human fibroblasts (BJ) and keratinocytes (HaCaT). Fibroblasts (ATCC^®^ CRL-2522 ™) and HaCaT (CLS–Cell Line Services GmbH, Germany) were obtained from the American Type Culture Collection (Manassas, VA 20108, USA). Both cell lines were maintained in DMEM (Modified Dulbecco’s Essential Medium, Corning) with L-glutamine, 4.5 g/L glucose, sodium pyruvate, supplemented with 10% FBS (fetal bovine serum, Gibco) and 1% antibiotics (100 U/mL penicillin and 1000 μg/mL streptomycin, Gibco). Cells were cultured in an incubator at 37 °C in a humid atmosphere of 95% air and 5% carbon dioxide (CO_2_). After the cells reached the appropriate confluence, the adherent cells were trypsinized with 0.25% trypsin/EDTA (Gibco) and seeded into 96-well plates. Cells, both fibroblasts and keratinocytes, were exposed to various concentrations (0.1%, 0.5%, 1% and 2.5%) of the tested surfactants (SLES, SAR, SLS, SDS) and ectoine separately or in combination of individual surfactants with 2.5% ectoine. Test compounds were dissolved in DMEM medium.

#### 3.2.5. Cell Viability Assay

In order to assess the viability of fibroblasts and HaCaT cells treated with surfactants and ectoine in vitro, a neutral red uptake assay was performed. This test is based on the ability of living cells to bind neutral red dye in lysosomes. By changing the amount of dye bound by the cells, it is possible to assess the cytotoxic activity of the compounds tested in vitro.

Cells were seeded in 96 well plates at a density of 1 × 10^4^ cells/well with fresh DMEM medium. After 24 h of culture, the medium was replaced with various concentrations (0.1%, 0.5%, 1% and 2.5%) of the four tested surfactants and/or ectoine and cultured for 1 and 24 h. The control group were cells cultured in DMEM medium without the addition of test substances. After this time, both cell lines were incubated for 2 h with a neutral red dye (40 µg/mL) dissolved in FBS-free DMEM medium. Then the cells were washed twice with phosphate buffered saline (PBS) and 150 µL destain solution (EtOH/AcCOOH/H_2_O 2.50%/1%/49%) was added to each well. The plates were gently shaken for 15 min until a neutral red was extracted from the cells and formed a homogeneous solution. Neutral red dye uptake was determined by measuring the optical density (OD) of the eluted dye at 540 nm in microtiter plate reader spectrophotometer FilterMax F5 (Thermo Fisher, Waltham, Middx, USA). As part of the cytotoxic study, three independent experiments in four replications were carried out for each surfactant and/or ectoine concentration. Cell viability is presented as a percentage of the control value (100%), i.e., cells not treated with test compounds.

#### 3.2.6. Statistical Analysis

Obtained values were presented as a mean ± SD. Significant differences between obtained values were analyzed using StatSoft, Statistica 9.0 using One-way ANOVA and Tukey’s test. Differences were considered significant when *p* < 0.05. Statistically significant differences are marked on the charts with letters. Statistically significant differences were marked with different letters.

## Figures and Tables

**Table 1:** Formulations of the analyzed samples.

	Concentration [%]							
	E	SLS	SCS	SLES	SARKO	SLS + E	SCS + E	SLES + E
Aqua	Ad. 100							
Sodium lauryl sulfate	-	1.0	-	-	-	1.0	-	-
Sodium coco sulfate	-	-	1.0	-	-		1.0	-
Sodium laureth sulfate	-	-		1.0	-	-	-	1.0
Sodium lauroyl sarcosinate	-	-	-	-	1.0	-	-	-
Ectoine	2.50	-	-	-	-	2.50	2.50	2.50
Citric acid	To pH 5.5 ± 0.1							

## References

[1] Graf R., Anzali S., Buenger J., Pfluecker F., Driller H. (2008). The multifunctional role of ectoine as a natural cell protectant. Clin. Dermatol..

[2] Bownik A., Stępniewska Z. (2016). Ectoine as a promising protective agent in humans and animals. Arh. Hig. Rada. Toksikol..

[3] Driller H., Bunger J., Degwert J. (2001). The protective function of compatible solute ectoine on the skin cells and its biomolecules with respect to UV-radiation, immunosuppression and membrane damage. IFSCC Mag..

[4] Pastor J.M., Salvador M., Argandoña M., Bernal Y., Reinabueno M.L., Iborra J.L., Nieto J.J., Cánovas M. (2010). Ectoines in cell stress protection: Uses and biotechnological production. Biotechnol. Adv..

[5] Kempf B., Bremer E. (1998). Uptake and synthesis of compatible solutes as microbial stress responses to high-osmolality environments. Arch. Microbiol..

[6] Buommino E., Schiraldi C., Baroni A., Paoletti I., Lamberti M., De Rosa M., Tufano M.A. (2005). Ectoine from halophilic microorganisms induces the expression of hsp70 and hsp70B′ in human keratinocytes modulating the proinflammatory response. Cell Stress Chaperon.

[7] Harishchandra R.K., Sachan A.K., Kerth A., Lentzen G., Neuhaus T., Galla H.J. (2011). Compatible solutes: Ectoine and hydroxyectoine improve functional nanostructures in artificial lung a surfactants. Biochim. Biophys. Acta.

[8] Buenger J., Driller H. (2004). Ectoin: An effective natural substance to prevent UVA-induced premature photoaging. Skin Pharmacol. Physiol..

[9] Marini A., Reinelt K., Krutmann J., Bilstein A. (2014). Ectoine containing cream in the treatment of mild to moderate atopic dermatitis: A randomised, comparator-controlled, intra-individual double-blind, multi-center trial. Skin. Pharmacol. Physiol..

[10] Bownik A., Stepniewska Z., Skowroński T. (2015). Protective effects of ectoine on behavioral, physiological and biochemical parameters of *Daphnia magna* subjected to hydrogen peroxide. Comp. Biochem. Physiol. C Toxicol. Pharmacol..

[11] Brand-Williamis W., Cuvelier M., Berset C. (1995). Use of a free radical method to evaluate antioxidant activity. LWT Food Sci. Technol..

[12] Farn R.J. (2006). Chemistry and Technology of Surfactants.

[13] Dominguez J.G., Balaguer F., Parra J.L., Pelejero C.M. (1981). The inhibitory effect of some amphoteric surfactants on the irritation potential of alkylsulphates. Int. J. Cosmet. Sci..

[14] Rosen M.J. (2006). Surfactants and Interfacial Phenomena.

[15] Moore P.N., Puvvada S., Blankschtein D. (2003). Challenging the surfactant monomer skin penetration model: Penetration of sodium dodecyl sulfate micelles into the epidermis. J. Cosmet. Sci..

[16] Dasilva S.C., Sahu R.P., Konger R.L., Perkins S.M., Kaplan M.H., Travers J.B. (2012). Increased skin barrier disruption by sodium lauryl sulfate in mice expressing a constitutively active STAT6 in T cells. Arch. Dermatol. Res..

[17] Faucher J.A., Goddard E.D. (1978). Interaction of keratinous substrates with sodium lauryl sulfate. I. Sorption. J. Soc. Cosmet. Chem..

[18] Hall-Manning T.J., Holland G.H., Rennie G., Revell P., Hines J., Barratt M.D., Basketter D.A. (1998). Skin irritation potential of mixed surfactant systems. Food Chem. Toxicol..

[19] Nielsen G.D., Nielsen J.B., Andersen K.E., Grandjean P. (2000). Effects of industrial detergents on the barier function of human skin. Int. J. Occup. Environ. Health.

[20] Bujak T., Nizioł-Łukaszewska Z., Wasilewski T. (2019). Sodium lauryl sulfate vs. sodium coco sulfate. Study of the Safety of Use Anionic Surfactants with Respect to Their Interaction with the Skin. Tens. Surf. Det..

[21] Bujak T., Wasilewski T., Nizioł-Łukaszewska Z. (2015). Role of macromolecules in the safety of use of body wash cosmetics. Colloids Surf. B.

[22] Bujak T., Nizioł-Łukaszewska Z., Wasilewski T. (2018). Effect of Molecular Weight of Polymers on the Properties of Delicate Facial Foams. Tens. Surf. Det..

[23] Bujak T., Wasilewski T., Nizioł-Łukaszewska Z. (2019). Effect of molecular weight of polyvinylpyrrolidone on the skin irritation potential and properties of body wash cosmetics in the coacervate form. Pure Appl. Chem..

[24] Wasilewski T., Seweryn A., Bujak T. (2016). Supercritical carbon dioxide blackcurrant seed extract as an anti-irritant additive for hand dishwashing liquids. Green Chem. Lett. Rev..

[25] Nizioł-Łukaszewska Z., Osika P., Wasilewski T., Bujak T. (2017). Hydrophilic Dogwood Extracts as Materials for Reducing the Skin Irritation Potential of Body Wash Cosmetics. Molecules.

[26] Bujak T., Nizioł-Łukaszewska Z., Zań A. (2020). Amphiphilic cationic polymers as effective substances improving the safety of use of body wash gels. Int. J. Biol. Macromol..

[27] Moore P.N., Puvvada S., Blankschtein D. (2003). Role of the Surfactant Polar Head Structure in Protein− Surfactant Complexation: Zein Protein Solubilization by SDS and by SDS/C12En Surfactant Solutions. Langmuir.

[28] Pezron I., Galet L., Clausse D. (1996). Surface interaction between a protein monolayer and surfactants and its correlation with skin irritation by surfactants. J. Colloid Interface Sci..

[29] Deo N., Jockusch S., Turro N.J., Somasundaran P. (2003). Surfactant interactions with zein protein. Langmuir.

[30] Stańczyk M., Gromadzińska J., Wasowicz W. (2005). Roles of reactive oxygen species and selected antioxidants in regulation of cellular metabolism. Int. J. Occup. Med. Environ. Health.

[31] Merck KgaA (2003). Ronacare Ectoin: The Natural Cell Protection Factor. https://www.ulprospector.com/en/eu/PersonalCare/Detail/824/34445/RonaCare-Ectoin.

[32] Ananthapadmanabhan K.P., Moore D.J., Subramanyan K., Misra K., Meyer F. (2004). Cleansing without compromise: The impact of cleansers on the skin barrier and the technology of mild cleansing. Dermatol. Ther..

[33] Mukherjee S., Edmunds M., Lei X., Ottaviani M.F., Ananthapadmanabhan K.P., Turro N.J. (2010). Original Contribution: Stearic acid delivery to corneum from a mild and moisturizing cleanser. J. Cosmet. Dermatol..

[34] Dharmendra K.Y., Surendra K., Eun-Ha C., Sandeep C., Mi-Hyun K. (2019). Molecular dynamic simulations of oxidized skin lipid bilayer and permeability of reactive oxygen species. Sci. Rep..

[35] Niki E. (2015). Lipid oxidation in the skin. Free Radic Res..

[36] Kunte H.J., Lentzen G., Galinski E.A. (2014). Industrial Production of the Cell Protectant Ectoine: Protection Mechanisms, Processes, and Products. Curr. Biotechnol..

[37] Katsarou A., Davoy E., Xenos K., Armenaka M., Theoharides T.C. (2000). Effect of an antioxidant (quercetin) on sodium lauryl sulfate-induced skin irritation. Contact Dermatitis.

[38] Nimse S.B., Pal D. (2015). Free radicals, natural antioxidants, and their reaction mechanisms. RSC Adv..

[39] Zou T.-B., He T.-P., Li H.-W., Xia E.-Q. (2016). The Structure-Activity Relationship of the Antioxidant Peptides from Natural Proteins. Molecules.

[40] Marcuse E. (1960). Antioxidative Effect of Amino-Acids. Nature.

[41] Elias R.J., Kellerby S.S., Decker E.A. (2008). Antioxidant Activity of Proteins and Peptides. Crit. Rev. Food Sci. Nutr..

[42] Hwang H.-S., Winkler-Moser J.K., Doll K.M., Gadgil M., Liu S.X. (2019). Factors Affecting Antioxidant Activity of Amino Acids in Soybean Oil at Frying Temperatures. Eur. J. Lipid Sci. Technol..

[43] Rojas M., Miskolczy Z., Biczók L., Pavez P. (2019). Effect of amino acid addition on the micelle formation of the surface-active ionic liquid 1-tetradecyl-3- methylimidazolium bromide in aqueous solution. J. Phys. Org..

[44] Afanas I.B., Dorozhko A.I., Brodskii A.V., Kostyuk V.A., Potapovitch A.I. (1989). Chelating and free radical scavenging mechanisms of inhibitory action of rutin and quercetin in lipid peroxidation. Biochem. Pharm..

[45] Yuan C.L., Xu Z.Z., Fan M.X., Liu X.Y., Xie Y.H., Zhu T. (2014). Study on characteristics and harm of surfactants. J. Chem. Pharm. Res..

[46] Benoit J., Cormier M., Wepierre J. (1988). Comparative effects of four surfactants on growth, contraction and adhesion of cultured human fibroblasts. Cell Biol. Toxicol..

[47] Bigliardi P.L., Michael J., Herron R.D., Dahl M. (1994). Effects of detergents on proliferation and metabolism of human keratinocytes. Exp. Dermatol..

[48] Liou G.Y., Storz P. (2010). Reactive oxygen species in cancer. Free Radic. Res..

[49] Han M.J., Kim B.Y., Yoon S.O., Chung A.S. (2003). Cell proliferation induced by reactive oxygen species is mediated via mitogen-activated protein kinase in Chinese hamster lung fibroblast (V79) cells. Mol. Cells.

[50] Mizutani T., Mori R., Hirayama M., Sagawa Y., Shimizu K., Okano Y., Masaki H. (2016). sodium lauryl sulfate Stimulates the Generation of Reactive Oxygen Species through Interactions with Cell Membranes. J. Oleo Sci..

[51] Evans M.D., Griffiths H.R., Lunec J. (1997). Reactive Oxygen Species and their Cytotoxic Mechanisms. Adv. Mol. Cell Biol..

[52] Gloxhuber C., Klunstler K. (1992). Anionic Surfactants: Biochemistry, Toxicology, Dermatology.

[53] Mehlhorn R.J., Packer L. (1976). Inactivation and reactivation of mitochondrial respiration by charged detergents. Biochem. Biophys Acta.

[54] Prottey C., Ferguson T.F.M. (1976). The effect of surfactants upon rat peritoneal mast cells in vitro. Food Cosmet. Toxicol..

[55] Shalel S., Streichman S., Marmur A. (2002). The mechanism of hemolysis by surfactants: Effect of solution composition. J. Colloid Interface Sci..

